# Influence of dialysis membrane composition on plasma bisphenol A levels during online hemodiafiltration

**DOI:** 10.1371/journal.pone.0193288

**Published:** 2018-03-12

**Authors:** Sebastian Mas, Enrique Bosch-Panadero, Pedro Abaigar, Vanesa Camarero, Ignacio Mahillo, Esther Civantos, Didier Sanchez-Ospina, Alberto Ruiz-Priego, Jesus Egido, Alberto Ortiz, Emilio González-Parra

**Affiliations:** 1 Renal, Vascular and Diabetes Laboratory, IIS-Fundación Jimenez Diaz UAM, Madrid, Spain; 2 Spanish Biomedical Research Centre in Diabetes and Associated Metabolic Disorders (CIBERDEM), Madrid, Spain; 3 Division of Nephrology, Hospital Universitario de Burgos, Burgos, Spain; 4 Department of Biostatistics and Epidemiology, IIS-Fundación Jimenez Diaz UAM, Madrid, Spain; 5 Division of Nephrology and Hypertension, IIS-Fundación Jimenez Diaz UAM, Madrid, Spain; 6 Department of Medicine, UAM, Madrid, Spain; 7 Kidney Research Network (REDINREN), Madrid, Spain; University of Glasgow, UNITED KINGDOM

## Abstract

**Introduction:**

Bisphenol A (BPA) is an ubiquitous environmental toxin that is also found in dialyzers. Online hemodiafiltration (OL-HDF) more efficiently clears high molecular weight molecules, and this may improve BPA clearance. However, the BPA contents of dialysis membranes may be a source of BPA loading during OL-HDF.

**Methods:**

A prospective study assessed plasma BPA levels in OL-HDF patients using BPA-free (polynephron) or BPA-containing (polysulfone) dialyzers in a crossover design with two arms, after a run-in OL-HDF period of at least 6 months with the same membrane: 31 patients on polynephron at baseline were switched to polysulfone membranes for 3 months (polynephron-to-polysulfone) and 29 patients on polysulfone were switched to polynephron for 3 months (polysulfone-to-polynephron).

**Results:**

After a run-in OL-HDF period of at least 6 months with the same membrane, baseline pre-dialysis BPA was lower in patients on polynephron (8.79±7.97 ng/ml) than in those on polysulfone (23.42±20.38 ng/mL, p<0.01), but still higher than in healthy controls (<2 ng/mL). After 3 months of polynephron-to-polysulfone switch, BPA was unchanged (8.98±7.88 to 11.14±15.98 ng/mL, ns) while it decreased on the polysulfone-to-polynephron group (23.42±20.38 to 11.41±12.38 ng/mL, p<0.01).

**Conclusion:**

OL-HDF for 3 months with BPA-free dialyzer membranes was associated to a significant decrease in predialysis BPA levels when compared to baseline BPA levels while on a BPA-containing membrane.

## Introduction

Bisphenol A (BPA, 2,2-bis-(4-hydroxyphenyl) propane) is an environmental toxin containing aromatic rings with structural similarity with phenols, which is present in polycarbonate plastics, epoxy resins and some dialyzer membranes. BPA has biological activity as a chemical switch in endocrine processes, and may negatively impact reproduction, weight and development. The maximum safe oral intake is 4 μg/kg/day [1]

Several studies have linked BPA exposure to the development of obesity, insulin resistance, metabolic syndrome, diabetes, atherosclerosis, hypertension and pathological albuminuria [2, 3], [4], [5]. One of the arguments used by regulatory agencies for considering BPA safe in the general population is the almost complete urinary clearance. However, serum BPA levels increase with decreasing renal function and are highest in individuals on hemodialysis [6]. Therefore, the general safety of BPA in the general population cannot be extrapolated to the dialysis population, since BPA accumulates in chronic kidney disease (CKD) patients.

Our group recently found lower plasma BPA levels in patients on conventional hemodialysis on BPA-free dialyzers than in those on BPA-containing dialyzers [7]. Thus, dialyzer BPA content may contribute to BPA burden in conventional hemodialysis patients and these patients are more sensitive to BPA accumulation and potential toxicity due to the loss of the physiological BPA excretion mechanisms in urine [7]. These findings differed from the conclusion of a prior, short term study, probably due to the slow build-up of BPA accumulation [8].

Hemodialysis is based on the ability of molecules to diffuse across a semipermeable membrane. Online hemodiafiltration (OL-HDF) improves mid-to-large molecule clearance by combining diffusive and convective transport [9]. However, high flux dialyzers used in OL-HDF may contain BPA, thus potentially contributing to the BPA burden. Therefore, the relative contributions of the OL-HDF technique, the dialyzer composition or the follow-up time to changes in BPA levels remains to be characterized. Our objective was to prospectively assess the impact of the BPA contents of dialyzers on plasma BPA levels in OL-HDF patients.

## Materials and methods

### Clinical design and patient recruitment

The study was approved by the IIS-Fundación Jiménez Díaz Ethics Committee and the study was performed in accordance with the Declaration of Helsinki and the European Union Clinical Trial Directive (2001/20/EC). Patients were enrolled after providing written informed consent.

In a prospective 9-month, crossover study we compared BPA-free and BPA-containing dialyzers in 72 patients on OL-HDF. Dialyzers only differed in the BPA content in the membrane. Specifically, BPA-free high-flux polynephron (polynephron) membranes (Elisio, NiproCorp, Osaka, Japan) were compared with high-flux polysulfone (Helixone^®^) dialyzers that contain BPA (Fx80; Fresenius, Bad Homburg, Germany).

**Fig 1:**
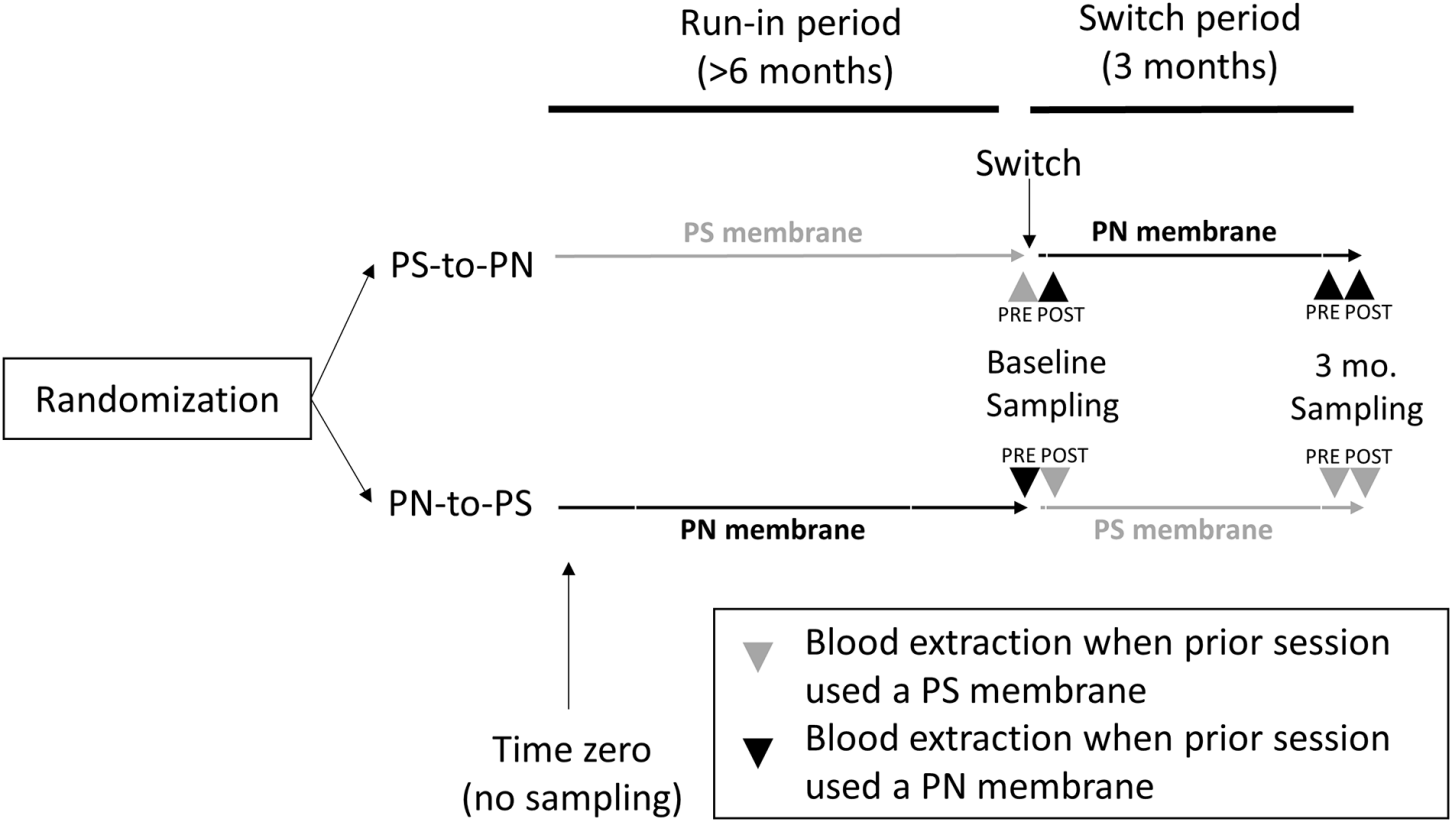
Study design: Timing of plasma sampling for BPA assessment. Baseline pre-dialysis plasma sampling took place after >6 months OL-HDF and prior to the first session with the switch dialyzer. Baseline post-dialysis sampling was obtained immediately after the first session with the switch dialyzer. Sampling was repeated pre- and post-dialysis after 3 months on the switch dialyzer.

**Fig 2:**
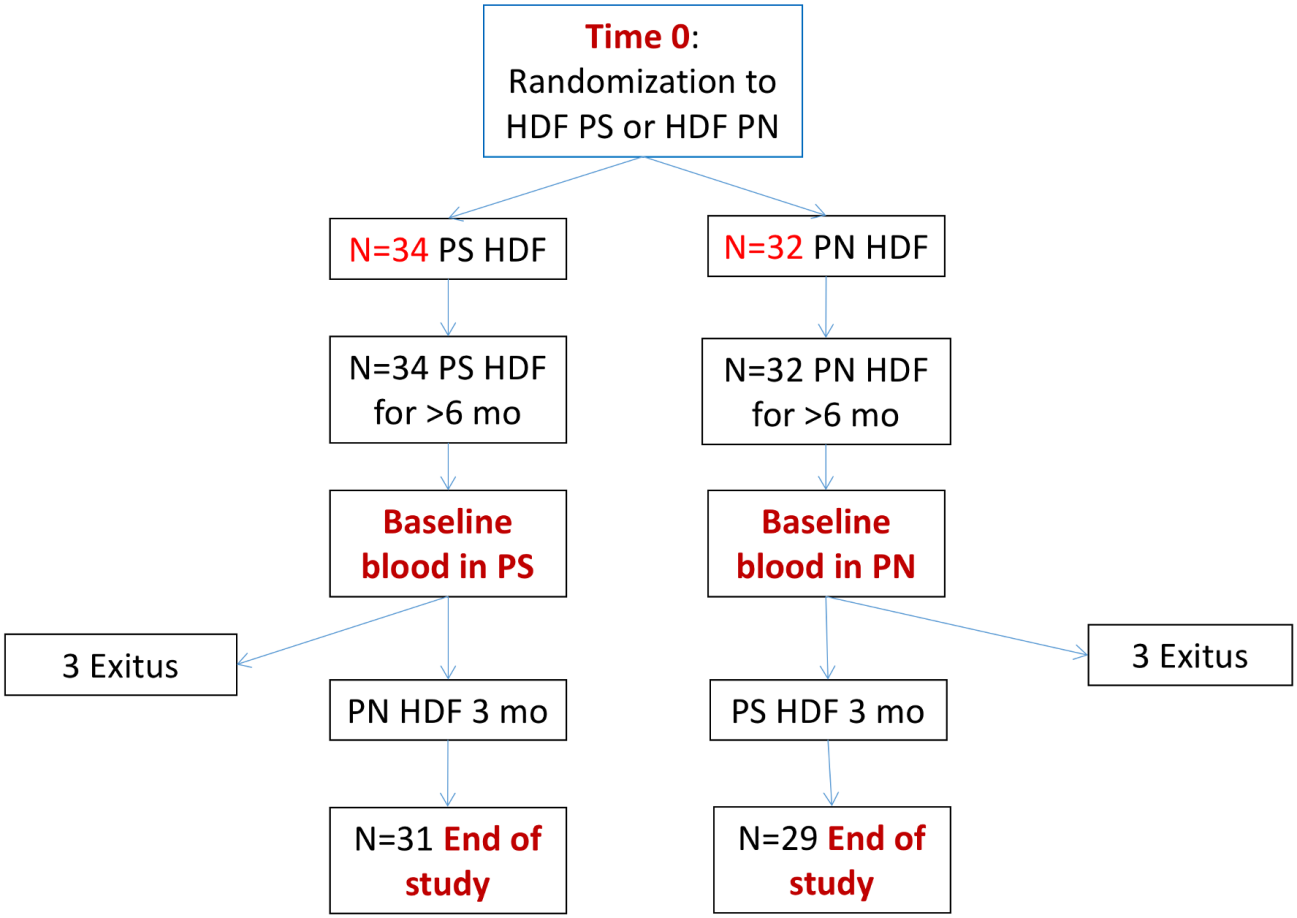
Patient flow chart.

At time zero (initiation of OL-HDF), patients were randomized into polynephron (n = 36) or.polysulfone (N = 36) dialysis membranes (Figs 1 and 2). No blood sampling was performed at this time point. This was followed by a run-in period of at least 6 months (Fig 1). Baseline blood sampling was performed 6 months after the last patient had been enrolled. At baseline, blood samples for BPA assessment were collected before (Pre-) the first OL-HDF session with the switch membrane and after (Post-) this first session with the switch membrane. Since this was a crossover study, we use the term switch membrane to denote the new membrane to which the patient was switched after baseline predialysis sampling. Thus, the switch membrane was polysulfone in polynephron-to-polysulfone patients and polynephron in polysulfone-to-polynephron patients. After three months on the switch membrane, the 3-month blood sample was obtained, both pre- and post-OL-HDF session with the switch membrane.

The primary end-point was change in pre-dialysis BPA between baseline and three months. The difference between baseline and three months could only be assessed in the 29 polynephron-to-polysulfone and 31 polysulfone-to-polynephron patients that completed the study and had a second sample for analysis (Fig 2). In this regard, the analysis should be considered per protocol and not intention-to-treat.

Blood lines were made of PVC (Fresenius Medical Care) in all cases. OL-HDF was performed with a blood flow >300 ml/min and dialysate flow 500 ml/min, using ultrapure dialysis fluids, defined as <0.1 CFU/ml and <0.03 EU/ml. The length of dialysis sessions, composition of dialysate and reinfusate (sodium 138–140 mmol/L, potassium 1.5–2.0 mmol/L, calcium 1.25–1.75 mmol/L, magnesium 0.5 mmol/L, chloride 106–109 mmol/L, bicarbonate 34–37 mmol/L, acetate 3–4 mmol/L, and glucose 1.0 g/L) was not modified during the study. All patients were on postdilution OL-HDF, with a minimum of 20 L of replacement volume per session. For on line production of replacement fluid, polysulfone BPA-containing ultrafilters (DIASAFE^®^plus; Fresenius, Bad Homburg, Germany) were used to prevent patient contact with endotoxins and other pyrogenic or bacteria-derived substances. Samples from individuals with normal renal function used as control samples (n = 10) were obtained from the blood bank after informed consent. Eighteen patients from the prior conventional hemodialysis study [7] were also enrolled in the current study.

### Clinical and biochemical variables

Fasting blood samples were drawn from the arteriovenous fistula just prior to a midweek dialysis session and plasma was frozen at –80°C. Blood was sampled before (predialysis) and after (postdialysis) a single dialysis session to determine BPA changes in one session using polynephron or polysulfone dialyzers. Laboratory parameters were assessed by automated blood analyzers (Advia 2400 chemistry system and Advia 2120 hematology system, Siemens).

### BPA assessment

BPA was measured by a high-sensitivity ELISA (Abnova), following the manufacturer’s instructions. The intra- and inter-assay coefficients of variation were 6.5% and 10.5%, respectively. This is the same technique used in the prior conventional hemodialysis study.

### Statistical analyses

Variables were described at baseline and at 3-month follow-up for each arm of the study, as mean and standard deviation. Comparisons between values were performed using paired sample t test, Mann-Whitney or Wilcoxon signed-rank test. All comparisons used the bilateral hypothesis test and a significance level of 0.05. One Way Anova was performed to compare pre and post-dialysis BPA at baseline and after 3 months. Correlations between BPA values and clinical variables were assessed using Spearman’s rank correlation coefficient.

## Results

### Clinical characteristics of patients in hemodiafiltration

Table 1 shows the clinical data of the patients at the initial at the baseline dialysis session and at 3 months after dialyzer switch. No significant differences were observed between baseline and 3 months for the parameters analyzed.

**Table 1:** Summary of clinical and biochemical variables.

	Polysulfone-to polynephron		Polynephron-to-polysulfone	
	Baseline	3 months	Baseline	3 months
**Kt/V urea**	1.71±0.05	1.78±0.05	1.71±0.03	1.71±0.03
**Qb (ml/min)**	384.5 ± 49.4	392.9 ± 48.7	377.2 ± 42.4	388.1 ± 47.1
**Dialysis time (min)**	225.2 ± 12.7	224.4±13.7	219.1 ± 16.5	216.7 ± 16.2
**Interdialysis weight gain (kg)**	1.9 ± 0.6	1.9±0.6	1.8 ± 0.8	2.0 ± 0.9
**Replacement volume (L)**	26.4 ± 3.8	26.0±3.8	25.9 ± 4.6	25.7 ± 4.3
**Substitution flow (mL/min)**	118.74±20.7	117.92±17.6	116.12±10.5	113.95±14.4
**Leukocytes (10**^**3**^**/μl)**	6196±420	6754±514	6500±387	6489±360
**Hb (g/dl)**	11.48±0.30	11.63±0.27	11.63±0.21	10.91±0.23
**25 OH vitamin D (ng/ml)**	11.37±1.32	14.39±1.64	12.19±1.36	14.34±1.68
**Total protein (g/dl)**	6.36±0.11	6.41±0.12	6.64±0.10	6.53±0.11
**Albumin (g/dl)**	3.56±0.09	3.60±0.10	3.74±0.07	3.76±0.11
**Ca (mg/dl)**	9.08±0.14	9.20±0.13	9.03±0.10	9.01±0.12
**P (mg/dl)**	4.16±0.20	4.15±0.24	4.43±0.23	4.65±0.28
**Cholesterol (mg/dl)**	150±6	147±6	150±5	146±6
**Triglycerides (mg/dl**)	112±9	106±10	102±11	105±8
**CRP (mg/L)**	20.77±5.54	16.65±6.11	16.32±4.20	21.36±9.18
**PTH (pg/dl)**	403±86	421±103	284±37	311±45
**Glucose (mg/dl)**	136±9	140±12	131±12	125±12
**BMI (Kg/m**^**2**^**)**	24.76± 0.73	24.82±0.73	25.87±0.89	25.93±0.88
**TSH (μUI/mL)**	2.72±0.49	2.93±0.59	2.17±0.35	2.36±0.36
**T3 (μg/dl)**	2.30±0.08	2.54±0.08	2.18±0.07	2.59±0.09
**T4 (μg/dl)**	1.18±0.04	1.21±0.03	1.11±0.04	1.14±0.04
**Cortisol (μg/dl)**	11.51± 1.39	10.88±1.17	10.80± 0.87	11.35±0.89
**Aldosterone (pg/dl)**	296.81±52.03	262.35±57.61	341.25±55.81	238.82±54.08

### Plasma BPA is lower in OL-HDF than in conventional hemodialysis patients

**Fig 3:**
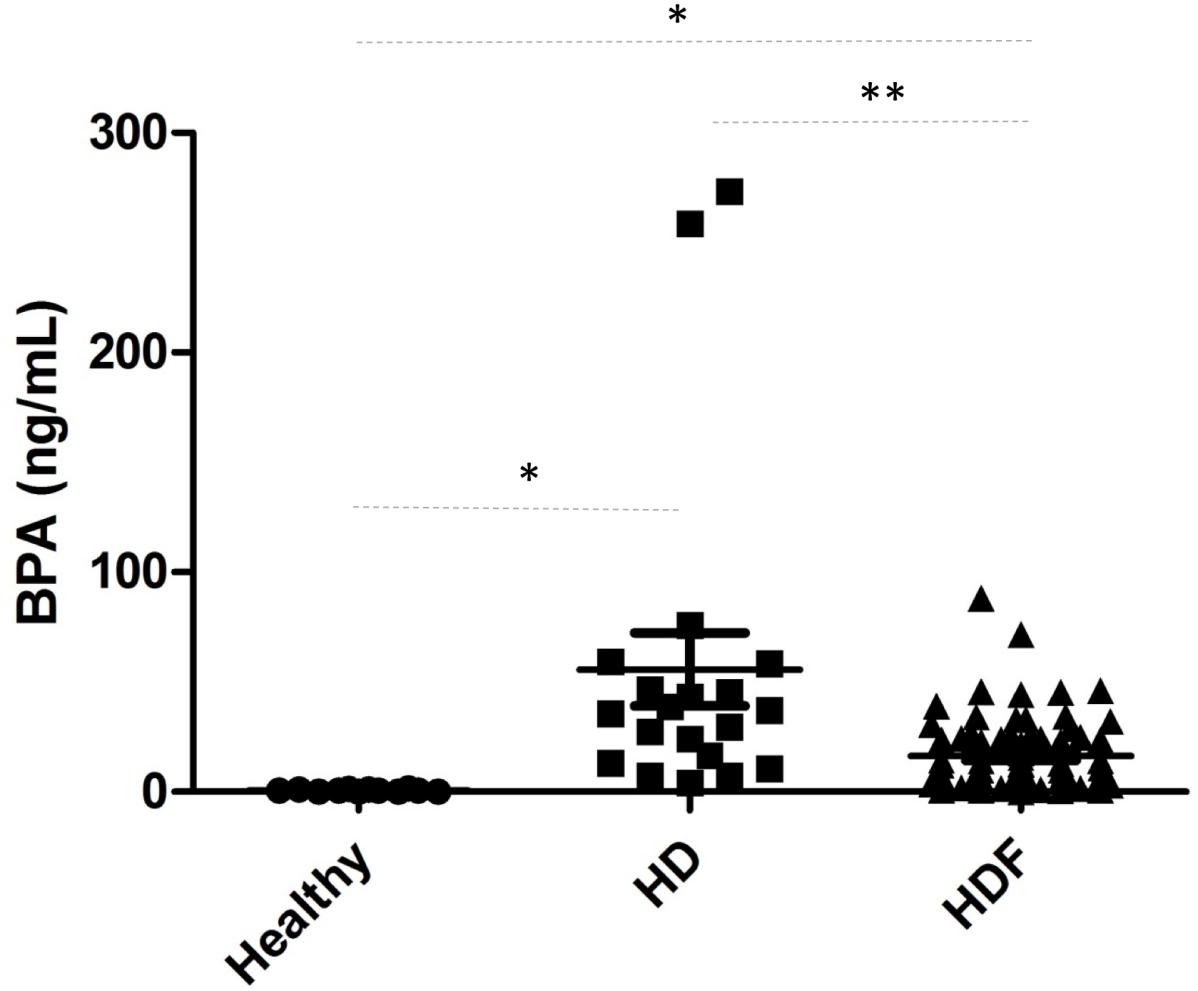
Plasma BPA concentration. Healthy subjects (n = 10); patients on online hemodiafiltration (HDF) (n = 58) and patients on conventional hemodialysis (HD) who were later enrolled in the present study (n = 18) [7]. Data expressed as mean±SD. For HD and OL-HDF, values correspond to baseline predialysis values in the switch studies. At this point, patients had been on HD or OL-HDF for >6 months. * p<0.05; ** p = 0.017.

We have now analyzed 60 patients that had been on OL-HDF using the same membrane for more than 6 months. After more than 6 months on OL-HDF with the same membrane, predialysis BPA concentration was 12.12±15.9 ng/ml (Fig 3). In 18 patients who participated in a prior study while on hemodialysis [7] before switching to OL-HDF for enrollment in the current study, plasma BPA fell from 98.96±120.75 ng/ml while on conventional hemodialysis to 13.14±13.78 ng/ml (p = 0.017) after more than 6 months on OL-HDF with the same membrane. By membrane, BPA fell from 52.73±60.6 to 6.78±6.48 in patients on polynephron and from 163.03±155.84 to 19,02±14.46 in patients on polysulfone, although this group of patients were randomized in switching to HDF.

Healthy individuals have values usually below the limit of quantification of the assay (2 ng/mL) (Fig 3). In this regard, plasma BPA was 6-fold (p <0.01) and 47-fold (p <0.01) higher in OL-HDF or conventional hemodialysis patients, respectively, than in the control population, confirming the accumulation of BPA in patients in end-stage kidney disease (ESRD).

The BPA concentration in replacement fluid was under the limit of detection of assay.

### Bisphenol A at baseline and after 3-month cross-over

**Fig 4:**
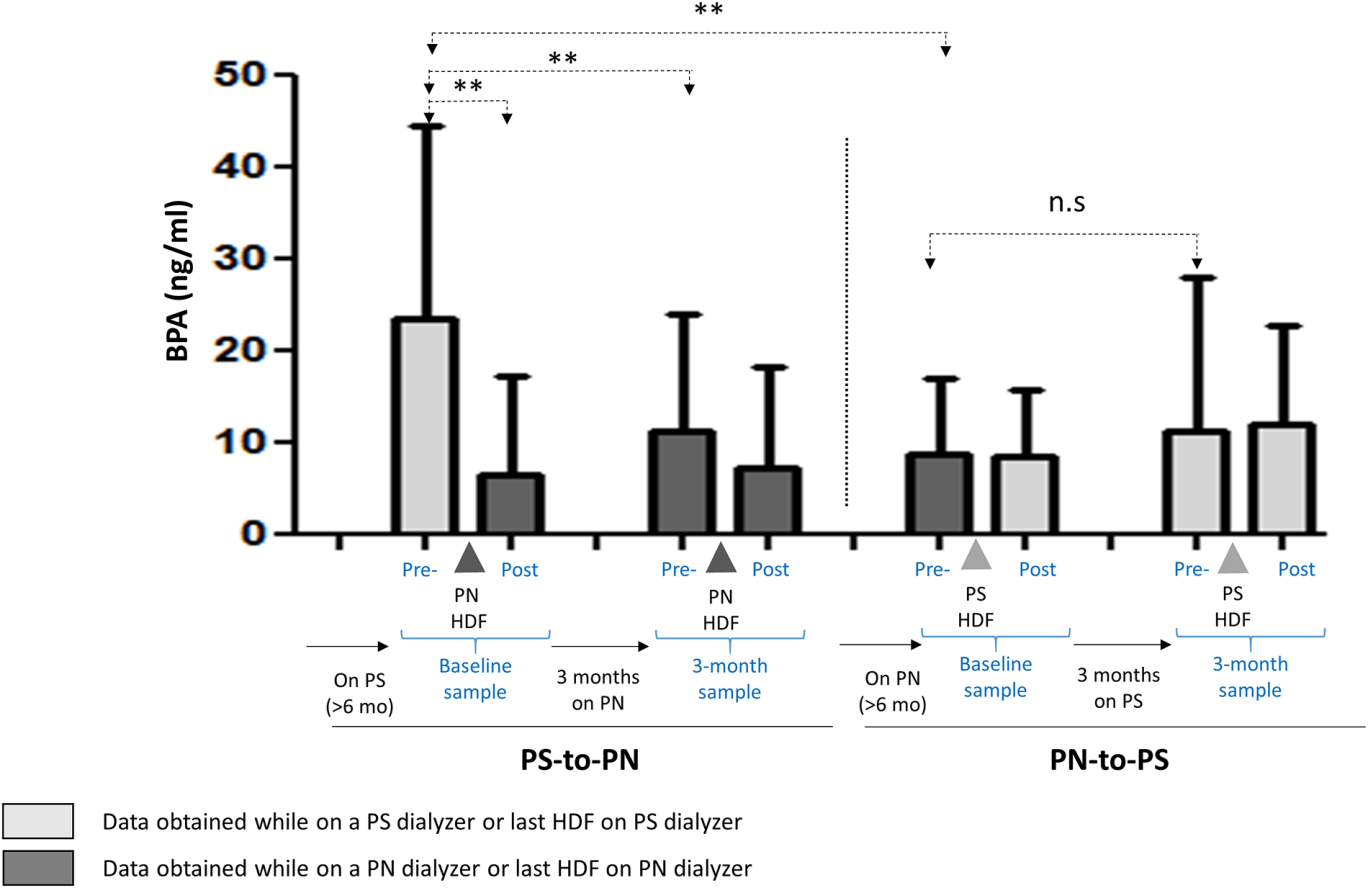
Plasma BPA concentration in patients on OL-HDF with polynephron (PN) or polysulfone (PS) membranes. Pre- and post-dialysis measurements are shown for the first (baseline) and the last (3-month) session after the switch. Prior to the baseline pre-dialysis sample, patients had been on OL-HDF with the opposite membrane for >6 months. Thus, baseline pre-dialysis values represent values corresponding to >6 months OL-HDF with the opposite membrane and were used as baseline values for the switch study, while baseline post-dialysis values were already obtained after the first session with the switch membrane. Data expressed as mean±SD. ** p<0.01. Pre- and Post- refers to pre-dialysis and post-dialysis (OL-HDF) session.

Predialysis baseline levels (blood sampled before the first dialysis session with the switch dialyzer, Fig 1) vary according to the dialyzer that had been used for >6 months prior to the first session with the switch dialyzer (Fig 4). In samples obtained just before the first session with the switch dialyzer, mean BPA levels were 23.42±20.88 ng/mL for patients using polysulfone dialyzers for >6 months, and 8.98±7.88 ng/mL for patients using polynephron for >6 months (p<0.01) (Fig 4).

Similarly, 3 months after dialyzer cross-over, BPA levels in polynephron-to-polysulfone patients change from 8.98±7.88 to 11.14±15.98 ng/mL (ns), while in polysulfone-to-polynephron patients, BPA decreased from 23.42±20.88 to 11.42±12.38 ng/mL (p<0.01), a 51% decrease (Fig 4).

### Pre-dialysis and post-dialysis plasma BPA concentration during a single OL-HDF session

The accumulation or elimination of BPA in a single OL-HDF session depends on the balance between membrane-leaked BPA and BPA clearance by OL-HDF. In OL-HDF with BPA-free membranes, only BPA clearance is expected to occur. In the first dialysis session with polynephron membranes following the switch from polysulfone was associated with a decrease in BPA levels from 23.42±20.88 ng/mL (pre-dialysis) to 6.44±10.77 ng/mL (post-dialysis), a 72% reduction (p <0.01). By contrast, the first dialysis with polysulfone following the switch from polynephron did not reduce BPA (pre-dialysis 8.98±7.88 to post-dialysis 8.43±6.99 ng/mL, ns), suggesting that the BPA load from the polysulfone membrane may have partially offset BPA clearance by OL-HDF (Fig 4).

After 3 months on polynephron dialyzers, the predialysis serum BPA levels (11.42±12.88 ng/mL) tended to decrease change post-dialysis (7.23±10.98 ng/mL, ns). In patients on polysulfone dialyzers for 3 months, no difference was observed between pre- and post-dialysis samples (11.14±15.98 to 11.86±10.41 ng/mL) (Fig 4).

### BPA levels do not correlate with other laboratory variables

Major laboratory and anthropometric variables associated to hemodialysis, nutritional and inflammatory status were collected. Correlations between BPA values and these variables were assessed using Spearman’s rank correlation coefficient. In general, plasma BPA was not associated to laboratory variables (S1 Table).

## Discussion

The main finding of the present study is that in patients on long-term BPA-containing dialyzers, a switch to a BPA-free dialyzer for three months was associated with a decrease in predialysis plasma BPA levels that was not observed in patients switched to BPA-containing dialyzers. Additionally, long-term (>6 months) use of BPA-free dialyzers was associated with lower pre-dialysis BPA levels that long-term use of BPA-containing dialyzers.

BPA is a 228 KD protein-bound molecule that accumulates in the circulation and intracellularly in CKD patients and may contribute to CKD manifestations [10]. BPA has been described to be 95% bound to proteins with an apparent dissociation constant of 2000 nmol/ml ex vivo [11]. The decrease of 72% in BPA levels during the first OL-HDF session when the switch membranes were BPA-free membranes is larger than expected for a protein-bound molecule. However, it is consistent with results from Quiroga et al. who observed an average reduction of 45% when dialyzers with and without BPA were analyzed as a single group [12]. This is consistent with a 72% reduction with BPA-free membranes and no change with BPA-containing membranes in our study (the mean of a 72% reduction with BPA free membranes and a 1% with BPA containing membranes is a 36% reduction, in the range of the 45% observed by Quiroga et al). This is in contrast to an average reduction ratio during OL-HDF of 5 to 10% for protein-bound uremic toxins [9]. In this regard, the factors modulating the reduction in BPA levels and BPA protein binding are not well understood. A key difference with other protein-bound toxins is that BPA can actually be released into the circulation by certain membranes during dialysis. However, this factor is not active in patients dialyzed with BPA-free membranes.

OL-HDF has the potential to increase serum BPA due to leaching from BPA-containing tubing or membranes used in the online production of reinfusion fluids. However, none of these factors differed between the two arms of the study. Additionally, BPA release from tubing is considered negligible and BPA was undetectable in the reinfusion fluid. While online production of dialysate solutions in the present study was associated to filtering the fluid through two BPA-containing polysulfone ultrafilters [13], the observation of a BPA-free replacement fluid is consistent with the observation that BPA leach from dialyzers is higher when blood, rather than saline, is filtered [14]. This has been ascribed to the presence in blood of hydrophobic components such as lipids and lipoproteins. BPA may also leach from BPA-containing dialyzer membranes during OL-HDF [6, 14]. However, no prospective, long-term studies have assessed the effect of chronic OL-HDF using state-of-the-art dialyzers with different BPA contents. In the present study, BPA levels were much lower in patients on OL-HDF than in patients that had participated in a conventional hemodialysis study in whom BPA was measured by the same assay (), independently of the dialysis membrane used. Thus, BPA levels were higher in the 69 patients in the hemodialysis study (64.55±93.8 ng/ml) [7] and also in the 18 patients in the hemodialysis study that went on to be enrolled in the present study (98.96±120.75 ng/ml) than in the patients treated for longer than 6 months with OL-HDF (23.42±3.81 ng/mL). The data support the concept advanced by Quiroga et al [12], that he dialysis technique (OL-FDF vs hemodialysis) indeed results in lower BPA levels. The BPA concentration range differed in both studies, probably due to the use of a different BPA assay, to individual patient or dialysis characteristics, to different duration of the study or to the existence of limitations to BPA clearance such as continuous leakage from dialyzer membranes (in the prior studies both BPA-containing and BPA-free membranes were grouped together). The short-term nature of Quiroga´s study using a heterogeneous group of dialyzers regarding the BPA contents of the dialyzer membranes did not allow the assessment of the relative contributions of the OL-HDF technique, the dialyzer composition or the follow-up time to the observed reduction in BPA levels [12]. Our data now point out that dialysis membrane composition is a key determinant of BPA levels in OL-HDF patients. In this regard, the switch design allowed an accurate assessment of the impact of dialysis membrane composition on plasma BPA levels in OL-HDF patients, and results are consistent with the prior hemodialysis study [7].

Additionally, BPA levels were even lower in OL-HDF patients using BPA-free dialyzer membranes for >6 months. From the available data, we cannot differentiate between BPA accumulation during the >6 month run-in with BPA-containing membranes from pre-existent high BPA values, since no sampling took place at time zero.

We found no relationship between BPA levels and analytical data. This may be related to the lower levels of BPA in OL-HDF than in conventional hemodialysis or to a low number of patients, given the complexity and heterogeneity of hemodialysis patients, or to a genuine lack of a relationship. In this regard, the study was designed to assess BPA levels, not to explore clinical or biochemical correlates.

This study has some limitations. The number of patients was low and baseline assessment of BPA concentrations was considered to represent the impact of >6 months of therapy with each membrane retrospectively. However, no samples were available from time zero (at the start of the >6 month run-in period).

In conclusion, in OL-HDF patients, BPA-free dialyzers are associated with a long-term larger decrease in BPA levels than the use of BPA-containing dialyzers, although BPA values at three months did not differ between the groups. Furthermore, our results support the concept that OL-HDF is associated with lower BPA levels than conventional high flux hemodialysis, although in OL-HDF, BPA still remains well above healthy control values. Further studies should explore the health consequences of BPA accumulation from dialysis membranes in patients on long-term OL-HDF.

## Supporting information

S1 Table Spearman´s rank correlation coefficient between plasma BPA levels and laboratory and anthropometric variables for patients on OL-HDF. (DOCX)
