# Supplementary material for: Influence of dialysis membrane composition on plasma bisphenol A levels during online hemodiafiltration
Source: PLoS One. 2018 Mar 12;13(3):e0193288. doi: 10.1371/journal.pone.0193288 (PMC5846770; doi:10.1371/journal.pone.0193288)
Supplement: S1 Table — (DOCX) [file pone.0193288.s001.docx]

**S1 Table. Spearman´s rank correlation coefficient between plasma BPA levels and laboratory and anthropometric variables for patients on OL-HDF.** p value in brackets.

| **Variable** | **Baseline BPA** | **3 months BPA** |
| --- | --- | --- |
| **Kt/V urea** | 0.02 (0.911) | -0.03 (0.799) |
| **Leukocytes (x 10^3^/µl)** | -0.03 (0.818) | -0.11 (0.418) |
| **Hb (g/dl)** | 0.05 (0.693) | -0.06 (0.639) |
| **25 OH Vit D (ng/ml)** | -0.20 (0.128) | -0.15 (0.274) |
| **Total protein (g/dl)** | -0.22 (0.099) | -0.07 (0.623) |
| **Albumin (g/dl)** | -0.06 (0.660) | -0.09 (0.526) |
| **Ca (mg/dl)** | 0.06 (0.663) | 0.06 (0.639) |
| **P (mg/dl)** | -0.17 (0.208) | -0.06 (0.672) |
| **Cholesterol (mg/dl)** | 0.02 (0.877) | -0.05 (0.710) |
| **Triglycerides (mg/dl)** | -0.09 (0.491) | -0.08 (0.879) |
| **CRP (mg/dl)** | -0.01 (0.964) | 0.04 (0.758) |
| **PTH (pg/dl)** | 0.0 (1.000) | -0.20 (0.127) |
| **Glucose (mg/dl)** | 0.18 (0.171) | -0.10 (0.484) |
| **BMI (Kg/m^2^)** | -0.10 (0.482) | -0.15 (0.258) |
| **TSH (µUI/mL)** | 0.08 (0.533) | 0.15 (0.251) |
| **T3 (µg/dl)** | 0.13 (0.333) | -0.25 (0.066) |
| **T4 (µg/dl)** | 0.03 (0.823) | -0.05 (0.690) |
| **Cortisol (µg/dl)** | -0.01 (0.946) | 0.04 (0.768) |
| **Aldosterone (pg/dl)** | -0.15 (0.250) | 0.18 (0.175) |
